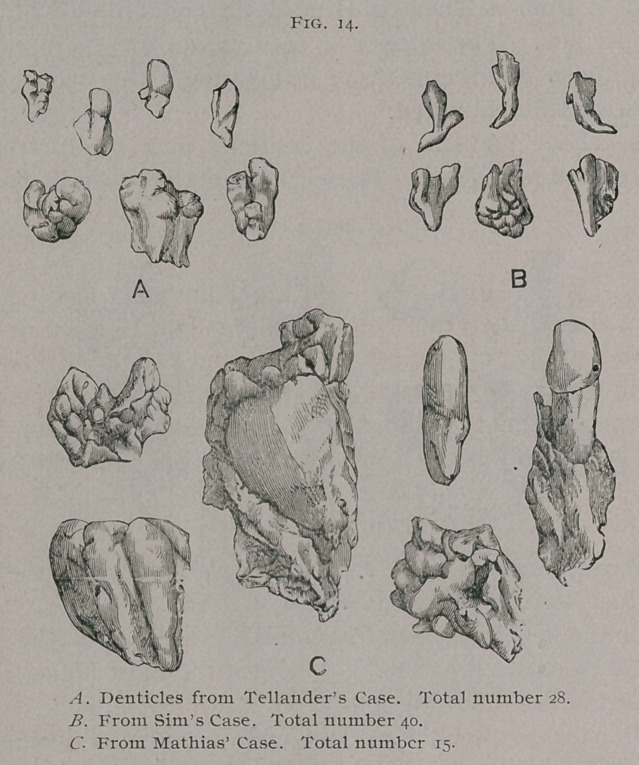# Odontomes

**Published:** 1890-01

**Authors:** J. Bland Sutton

**Affiliations:** Assistant Surgeon, Middlesex Hospital, London; Erasmus Wilson Lecturer, Royal College of Surgeons, England


### THE JOURNAL

OF

### COMPARATIVE MEDICINE AND VETERINARY ARCHIVES.

VOL. XI. JANUARY 1890. No. 1.

### ODONTOMES.

BY J. BLAND SUTTON, F.R.C.S.,

*Assistant Surgeon, Middlesex Hospital, London; Erasmus Wilson Lecturer, Royal College of Surgeons, England.*

*Def. Odontomes are neoplasms composed of dental tissues, in varying proportions and different degrees of development, arising from tooth-germs, or teeth still in the process of growth.*

IN mammals three distinct parts are concerned in the formation of a tooth: 1. The enamel organ. 2. The dentine papilla. 3. The tooth-follicle.

These parts are somewhat digrammatically represented in Fig. 1.

Using the parts concerned in the formation of a tooth as the basis of a classification, Odontomes may be arranged as in the subjoined table:—

A. ABERRATIONS OF THE ENAMEL ORGAN.
   *Epithelial Odontomes.*

B. ABERRATIONS OF THE FOLLICLE.
   *1. Follicular Cysts.*
   *2. Fibrous Odontomes.*
   *3. Cementomata.*
   *4. Compound Follicular Odontomes.*

C. ABERRATIONS OF THE PAPILLA.
   *Radicular Odontomes.*

### D. Aberrations of the Whole Tooth-germ.

### *Composite Odontomes.*

### *A.* Abberations of the Enamel Organ.

*Epithelial Odontomes.*—This group includes those tumors of the jaw termed by McEve[1] multilocular cystic epithelial tumor; they present the following characters:—

Fig. 1.

The parts concerned in a developing tooth.

Most of the patients come under observation at the age of twenty years,[2] although the disease may occur at any period from infancy to old age. More commonly the lower jaw is affected, and the molar region is usually, though not exclusively, involved.

In typical specimens the tumor displays on section a congeries of cysts, in size very various, but they rarely exceed an inch in diameter. The cysts are separated by thin, fibrous septa, in some cases by osseous tissue. The cavities, are, as a rule, filled with mucoid fluid of a brownish color. The growing portions of the tumor are of a reddish-brown color, not unlike that of a myeloid sarcoma.

Histologically, these tumors are composed of branching and anastomosing rods or columns of epithelium, portions of which form alveoli. The stroma is composed of fibrous tissue; when abundant, embryonic tissue in various stages is present. The cells occupying the alveoli vary; the outer layer may be columnar, whilst the central

Fig. 2.

An epithelial odontome.

[1] For a full account of these tumors see McEve's paper in *Trans. Odonto. Soc.*, Great Britain, xviii., p. 39, 1885.

[2] In horses odontomes usually occur at about adult age; *i. e.* 5–7 years; but in dogs are more frequent in old animals.—[Ed. note.]

cells degenerate and give rise to a reticulum of stellate cells resembling in structure the *stratum intermedium* of the enamel organ.

The naked eye appearance of these tumors is very characteristic. The specimen represented in Fig. 2 is preserved in the museum of University College; it was removed by Listow.

Falkson and Bryck believe these tumors to arise in persistent portions of epithelium forming the enamel organs. McEve suggests that they may arise from epithelial ingrowths around the alveoli, some of which posssibly represent teeth long since suppressed in the process of evolution.

*B.* ABERRATIONS OF THE FOLLICLE.

*Follicular odontomes.*—This includes those swellings usually called dentigerous cysts, but as the term has been loosely applied to any cyst which bears teeth, even ovarian dermoids, it will be well to alter the name of those connected with teeth to *follicular* cysts or odontomes.

FIG. 3.

The microscopical appearance of an epithelial odontome.

They arise in relation with teeth which have remained within the jaws—retained teeth; they are associated most commonly with the permanent set, but may affect also a temporary tooth; this, however, is rare. They are most frequently connected with the molars, and occur in the upper and lower jaws. In the latter situation they expand the bone and produce extensive deformity. In the upper jaw the cyst may invade the antrum.

A glance at the relation of a tooth to its follicle, (Fig. 4) will explain that if fluid accumulates between the tooth and follicle, a cyst will be formed, and its size will depend upon the amount of fluid. Charles Tomes suggests that follicular cysts are due to the excessive formation, around a retained tooth, between the enamel and the wall of the follicle, of the fluid which is normally found after the complete development of a tooth.

The thickness of the walls is very various; in some thin and crepitant, in others measuring half an inch or more. Surrounding a cavity containing viscid fluid and the fangs of a tooth; such teeth are often ill-developed. Sometimes the crown of the tooth is well-formed, but the fang is imperfect or truncated as in Fig. 5. In a few specimens the tooth is loose in the cavity; occasionally it is inverted and not infrequently wanting. The walls of follicular cysts always contain calcific matter; it varies considerably in amount.

FIG. 4.

A normal follicle *in situ*.

Follicular cysts may suppurate; in man this rarely occurs, but in other mammals it is by no means uncommon. The specimen sketched in Fig. 6 occurred in a prehensile-tailed porcupine. Each lower jaw was occupied by a cyst which had suppurated and caused the death of the porcupine.

FIG. 5.

A follicular cyst containing a bicuspid tooth with truncated fang. Museum R. Coll. of Surgeons.

FIG. S.

A follicular cyst developed in connection with the lower incisor of a porcupine *Sphingurus prehensilis.*

*Fibrous odontomes.*—In a developing tooth a portion of the connective tissue in which it is embedded is found to be denser and more vascular than the rest; it also presents a fibrillar arrangement. This condensed tissue is known as the tooth-sac, and when fully developed presents an outer firm wall and an inner loser layer of tissue. At the root of the tooth the follicle-wall blends with the dentine papilla, and is indistinguishable from it. Before the tooth cuts the gum it is completely enclosed within this capsule.

Under certain conditions this capsule becomes greatly increased in thickness and so thoroughly encysts the tooth that it is never erupted. Such thickened capsules are mistaken for fibrous tumors especially if the tooth is small and ill-developed. Under the microseope they present a laminated appearance with strata of calcific matter. To these, the term fibrous odontomes may be applied. They are more common in ruminants than in other mammals, and are especially frequent in goats. As a rule they are multiple, four being by no means an unusual number. I have met with them in marsupials, bears, lions, and they have been seen in the human subject.

Fig. 7.

A fibrous odontome.

There is good reason for the belief that rickets is responsible for some of these thickened capsules. It has long been known that in rickety children the teeth are late in appearing. Mr. Shaw many years ago attributed this to the diminution in the size of the jaws and increase in the size of the teeth. Several years ago I endeavored to point out that the delay must be attributed to abnormal thickness of the follicle. That the tooth-sac should thicken in rickety children need not surprise us when we remember that this remarkable disease affects most particularly those membranes engaged in the production of bone.

Such thickenings of the follicles occur in rickety children as the following specimen preserved in the museum of the R. Coll. Surgeons testifies. It is thus described in the catalogue: "Sections of two myeloid tumors developed symmetrically in the angles of the lower jaw. Their surfaces are covered by the external layer of compact tissue of the bone which they have expanded and thinned."

These tumors were removed by Mr. Heath from a boy seven-and-a-half years old, with rickety legs, but he was well nourished

[1] *Med. Chir. Trans.*, Vols. XVII. and XXVI.

when the tumors were removed. They began when he was a year-and-a-half old, and increased slowly and painlessly. He had a good deal of difficulty with his teeth.

I have carefully examined these specimens and have no hesitation in pronouncing them to be overgrown tooth follicles due to rickets. It must be remembered that a few myeloid cells are not sufficient to make a myeloid sarcoma; the giant cells should make up a very large proportion of the tumor.

FIG. 8.

The boy with symmetrical tumors of the mandible described in the text [after Heath].

The long history of the Case—six years—the general characters of the tumors, their symmetry, and the rickety constitution of the boy, strongly support the view that the tumors in this case are of the same nature as those in the bears, and goats. The curious square-shaped look of the boy's face, as shown in Fig. 8, is very characteristic, and was strongly marked in the case of the goats.

FIG. 9.

Fibrous odontome from an Assyrian Bear. (Natural size.)

It has been urged that in the fibrous odontomes we have a well-formed tooth, whereas odontomes are usually devoid of all resemblance to teeth, so far as shape is concerned. It is a remarkable fact that in the upper jaw odontomes often have a well-shaped tooth attached to them as in Hare's case, (see Fig. 16,) in the lower jaw they merely consist of a conglomeration of dental tissues. Fibrous odontomes in the lower jaw follow this rule and usually contain an ill-shaped denticle or denticles. It seems to me that this difference may be explained by the anatomical differences of the jaw bones. In the upper jaw they invade and luxuriate in the antrum, and attain a far larger size than is possible between the two resisting plates of the lower jaw.

*Cementomata.*—When the capsule of a tooth becomes enlarged as in the specimens just considered, and these thick capsules become ossified, the tooth will become embedded in a mass of cementum. To this form of odontome the name cementoma may be applied. Odontomes of this character occur most frequently in horses, and sometimes attain a large size. Broca[1] has described and figured specimens from horses. Mr. Charles Tomes[2] has described one weighing ten ounces, and I have given an account of one from a horse which weighed twenty-five ounces. The main portion of this odontome is sketched in Fig. 10. When divided, three teeth could be made out, embedded in cementum. The periphery of the tumor was cautiously decalcified in hydrochloric acid and sections then prepared for the microscope. The structure of the decalcified mass was very instructive for the periphery of the tumor exhibited the laminated disposition seen in fibrous odontomes.[3]

FIG. 10.

A Cementoma from a Horse.

Among classical odontomes, the most typical specimen, which illustrates well the characters, macroscopical and microscopical, of a cementoma is that recorded by Dr. Forget from the lower jaw of a man aged forty. Before operating on the tumor, Maisonneuve, in whose practice the case occurred, determined to extract a decayed molar, and the odontome came away with it. Under the microscope the tumor, which was of the size of a pigeon's egg, consisted of cementum only (Fig. 11.) Broca[4] gives a drawing representing a vertical section through this odontome. The

[1] *Traité des Tumeurs.*
[2] *Trans. Odont. Soc.* of Gt. Britain.
[3] *Trans. Odont. Soc.* of Gr. Britain, 1887, contains a full description of this odontome.
[4] *Traité des Tumeurs*, Tom. ii.

shape, disposition, and structure of this odontome allow no escape from the conclusion that it had its origin in an overgrown, but ossified, dental follicle.

FIG 11.

*A*. Cementoma adherent to a human molar tooth. (After Forget.)

*B*. A section through the same. (After Broca.)

This opinion is further strengthened by the evidence furnished by Magitôt[1] after a re-examination of the specimen. This observer detected by the microscope two prolongations of dentine

FIG. 12.

A compound follicular odontome from a Thar *Capra jemlaica*. *N*. Superior maxillary division of the fifth nerve.

which seemed to form the boundary of a large cavity ; this he regarded as the remains of the pulp chamber.

*Compound Follicular Odontomes*.—If the thickened capsule

[1] *Traité des Anomalies du Systeme Dentaire*, Plate xix. Fig. 2.

ossify sporadically instead of *en masse* a curious condition is brought about, for the tumor will then contain a number of small teeth or denticles consisting of cementum, or dentine, or even ill-shaped teeth composed of three dental elements, cementum, dentine, and enamel. The number of teeth and denticles in such tumors vary greatly, and may reach a total of three or four hundred. The odontome sketched in Fig. 12, was of this nature, I obtained it from a Thar or Himalayan goat which had one in each upper jaw. The interior of each tumor was occupied with teeth, denticles and fragments of cementum of varying size, numbering in all three hundred.

The shape and size of the denticles may be inferred from those sketched in Fig. 13. These fragments were firmly embedded

FIG. 13.

Denticles from the odontome of the Thar.

in the fibrous walls of the tumor whilst those which were free in the sac had become loosened by suppuration.

Tumors of this character have been described in the human subject by several observers. Amongst the most noteworthy we may mention the following :—

Tellander, of Stockholm, met with a case in a woman aged twenty-seven years. The right upper first molar, bicuspids and canine of the permanent set had not erupted, but the spot where these teeth should have been was occupied by a hard, painless enlargement, which the patient had noticed since the age of twelve years. Subsequently this swelling was found to contain minute teeth. There were nine single teeth, each one perfect in itself, having a conical root with a conical crown—tipped with enamel ; also six masses built up of adherent single teeth. The denticles presented the usual characters of supernumerary teeth. About a year afterwards a tooth was found making its appearance in the spot from which the host of teeth was removed. A few of the teeth are represented in Fig. 14.

A similar case has been recorded by Mr. John Tomes, the details of which were communicated to him by Mr. Mathias,[1] whilst on medical service in India. A Hindoo, aged twenty, had a large number of ill-formed teeth united. Further search was instituted until at last fifteen masses of supernumerary teeth

FIG. 14.

*A*. Denticles from Tellander's Case. Total number 28.
*B*. From Sim's Case. Total number 40.
*C*. From Mathias' Case. Total number 15.

and bone were removed. The soft parts rapidly healed, the deformity disappeared ; the only peculiarity noticeable was the absence of the central and lateral incisors. The canines occupied their usual position. A few of the fragments are shown in Fig. 14, *C*.

A third example of this remarkable condition has been recorded by Professor Windle and Mr. Humphreys.[2] The case occurred in the practice of Mr. Sims at the Dental Hospital, Birmingham. The tumor was found in the mouth of a boy aged ten

[1] *Trans. Odonts Soc. Great Britain*, vol. iii, p. 365.
[2] *Journal of Anat. and Physiology*, vol. xxi, 1887.

years. It was found that neither the deciduous nor permanent right lateral incisor or canine had erupted. The space thus unoccupied was filled by a tumor with dense unyielding walls which occasioned no discomfort. On opening this cyst forty small denticles of curious and irregular forms were removed from the interior. Some of the denticles are represented in the accompanying drawing, Fig. 14, *B*, of their exact size. The largest possessed fourteen cusps. Many are caniniform, with fairly well-formed crowns and roots, the former being covered with enamel. Some resembled supernumerary teeth, while others consisted of several small denticles cemented together.

Heath[1] records briefly a case in which a boy aged four years had a cyst connected with the jaw in the situation of a temporary canine. The cyst contained seven small irregular nodules of dentine and enamel.

Mr. H. L. Albert[2] met with the following case. A schoolboy, aged fourteen years had a swelling about the size of a marble over the upper left canine and bicuspid; from this swelling a supernumerary tooth made its appearance, and was extracted; fourteen days later another tooth appeared, this was removed. Three weeks afterwards a third tooth was erupted and extracted, and at the same time a piece of enamel fell from the tumor; subsequently a fifth denticle appeared and fell out of its own accord. Broca[3] removed from the lower jaw of a child, two years and-a-half old, a tumor composed mainly of fibrous tissue. In its interior nodules of dentine coated with cementum and enamel were found.

Logan[4] has described a cyst from the upper jaw of a horse, containing more than four hundred denticles varying from a millet seed to teeth an inch-and-a-half in length. They seemed to sprout from the cyst wall.

[1] Ashhurst *Ency. of Surgery.*

[2] *Illustrated Med. Journal*, August 10, 1889.

[3] *Traite des Tumeurs*, Paris 1887. Magitot figures this specimen in *Anomalies du Systeme Dentaire.*

[4] JOURNAL OF COMP. MED. AND SURGERY. New York, October. 1887.

[TO BE CONTINUED.]

## Figures and Tables

**Fig. 1. f1:**
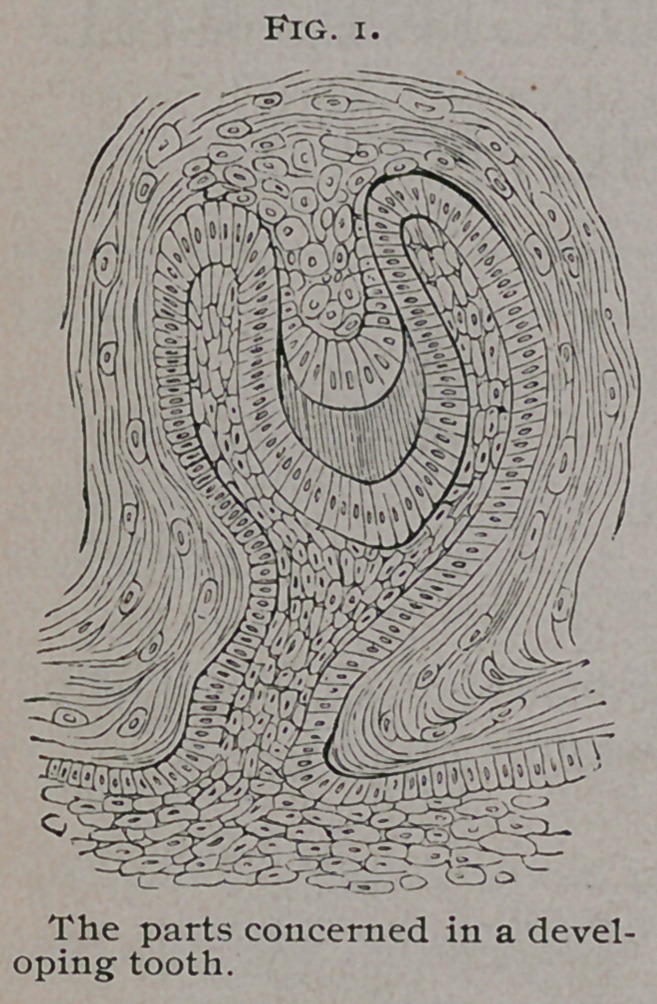


**Fig. 2. f2:**
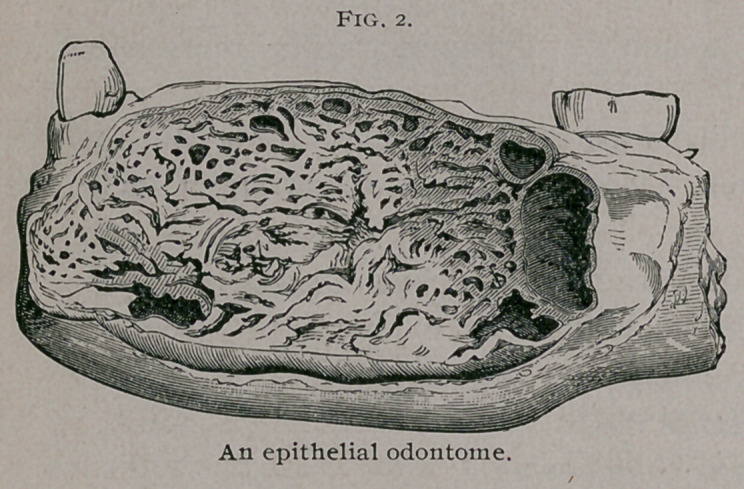


**Fig. 3. f3:**
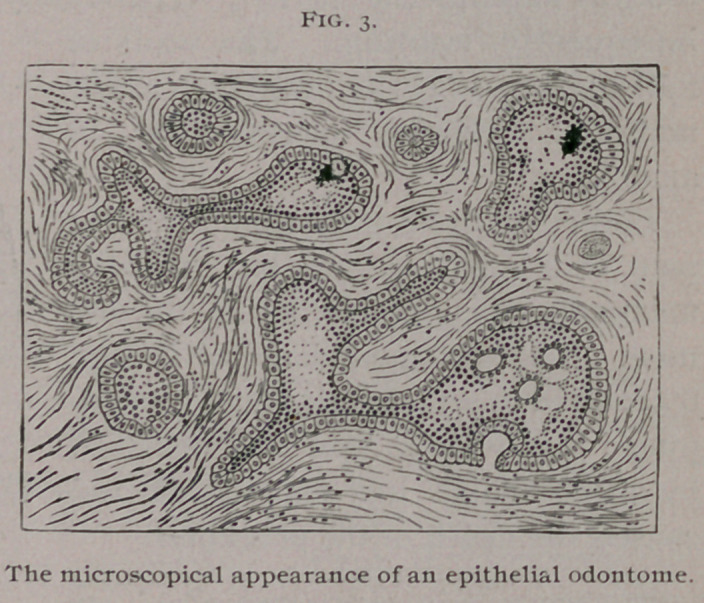


**Fig. 4. f4:**
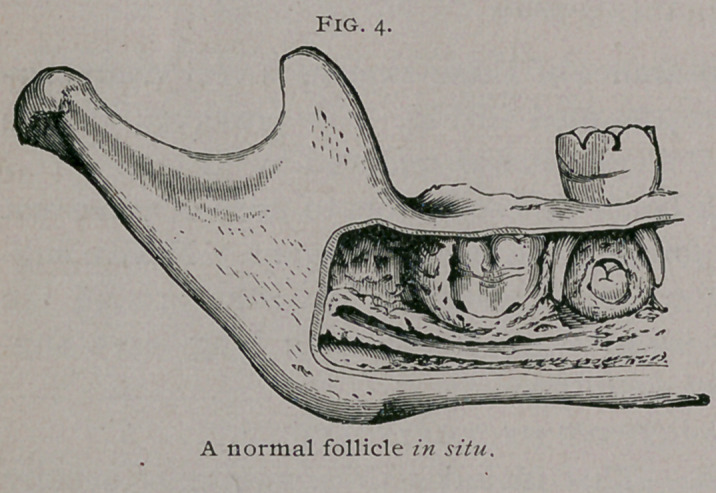


**Fig. 5. f5:**
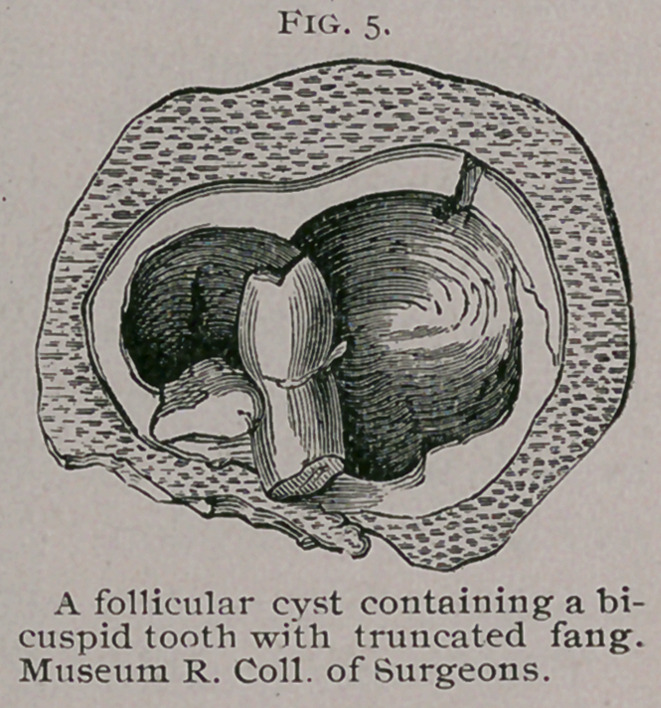


**Fig. S. f6:**
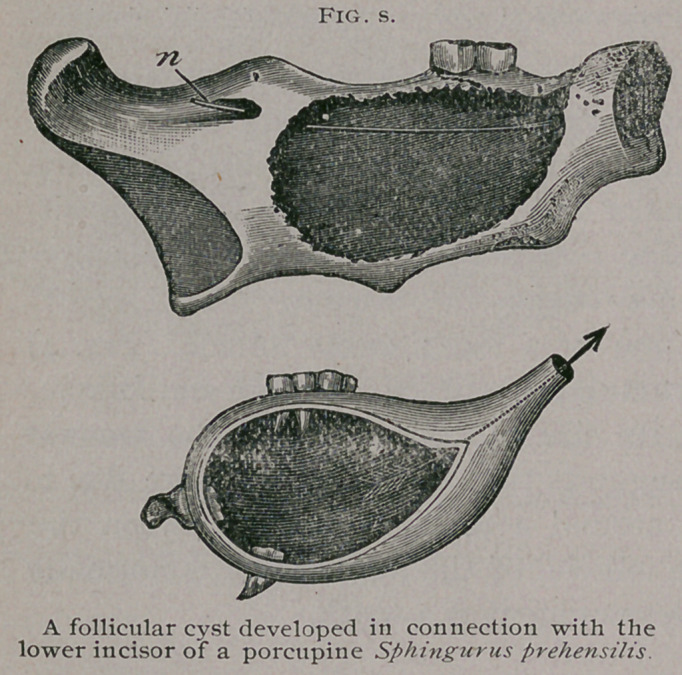


**Fig. 7. f7:**
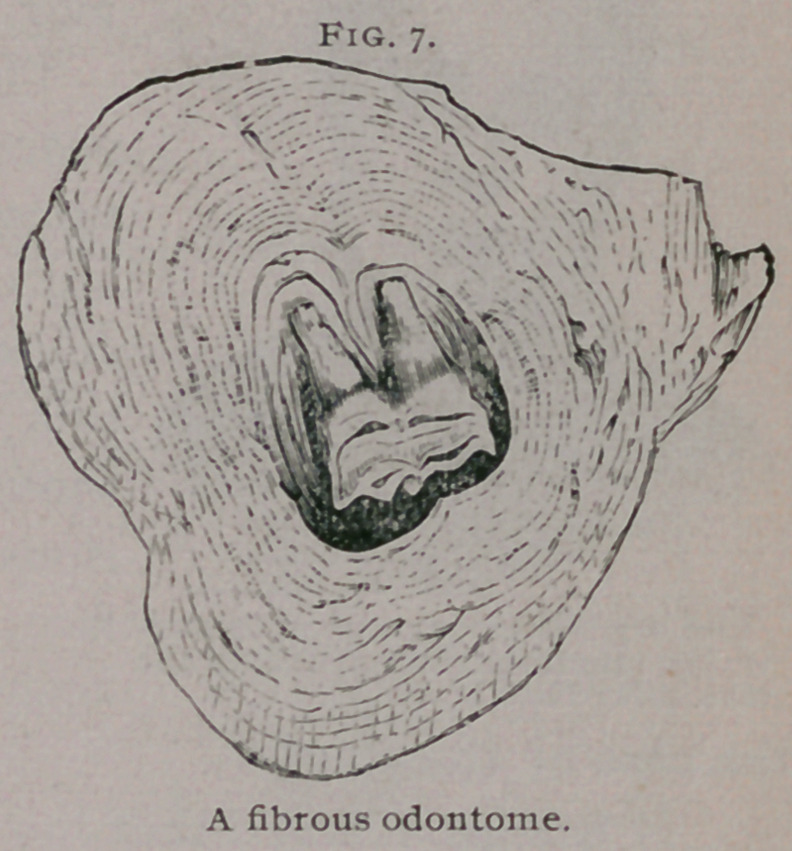


**Fig. 8. f8:**
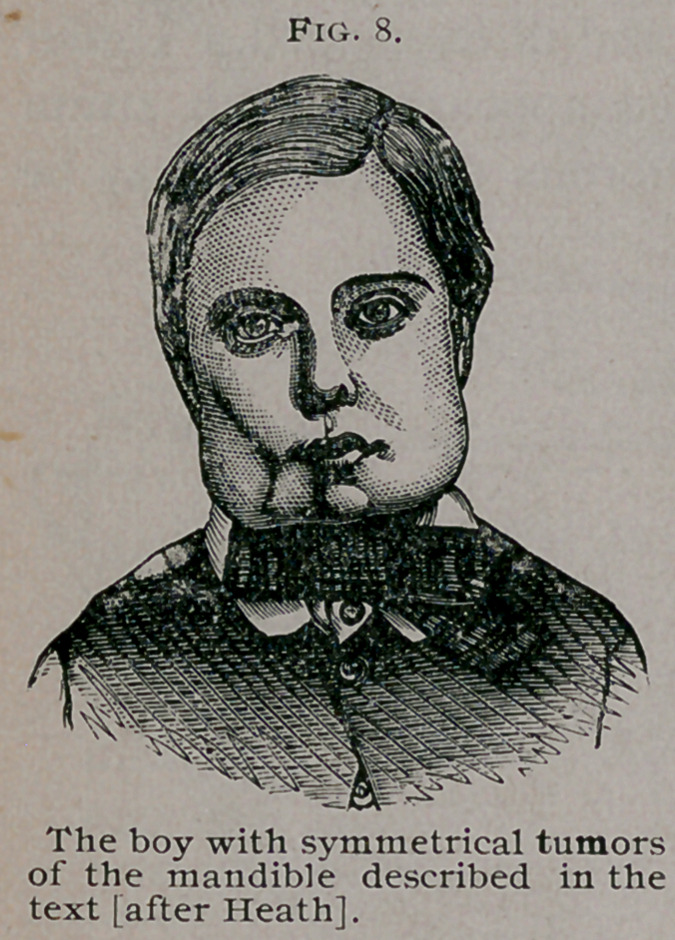


**Fig. 9. f9:**
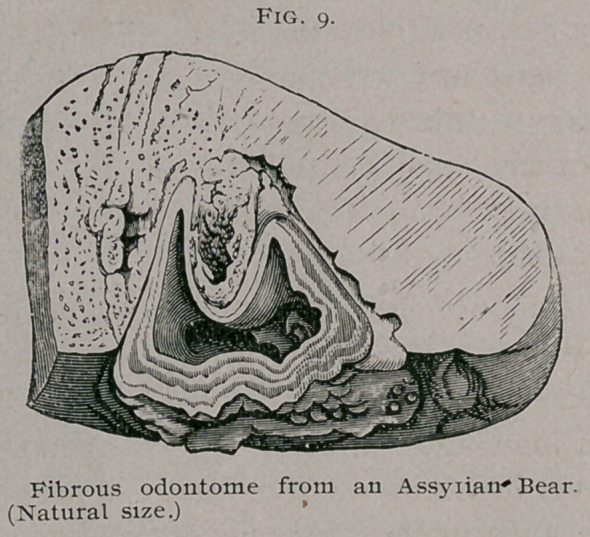


**Fig. 10. f10:**
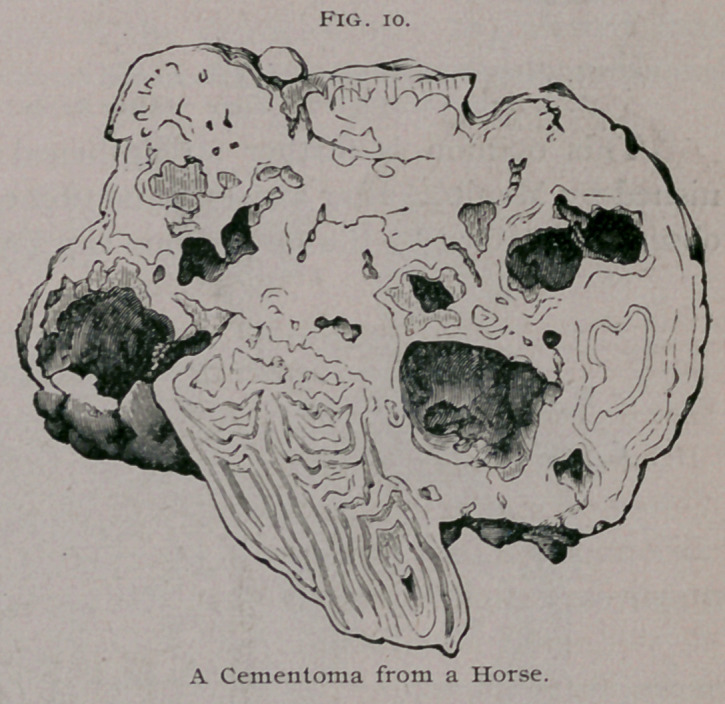


**Fig. 11. f11:**
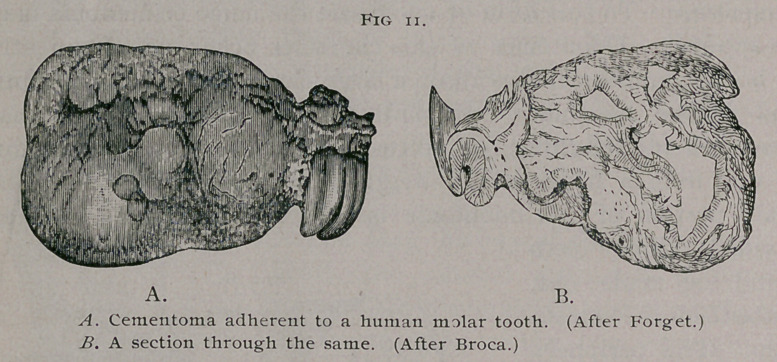


**Fig. 12. f12:**
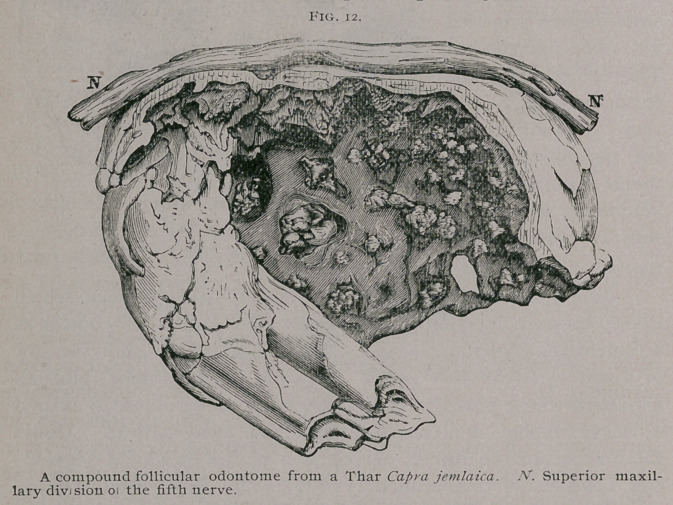


**Fig. 13. f13:**
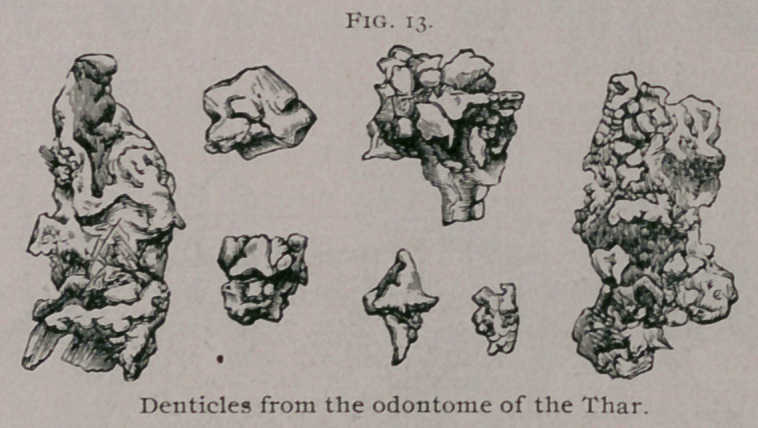


**Fig. 14. f14:**